# Supplementary material for: Comparison of Three Diagnostic Definitions of Metabolic Syndrome and Estimation of Its Prevalence in Mongolia
Source: Int J Environ Res Public Health. 2023 Mar 11;20(6):4956. doi: 10.3390/ijerph20064956 (PMC10048927; doi:10.3390/ijerph20064956)
Supplement: Supplementary file 1 [file ijerph-20-04956-s001.zip › ijerph-2209466-supplementary.pdf]

Supplement Figure S1. Comparison of the distribution of metabolic syndrome in individual metabolic syndrome components by three definitions. Each mosaic plot shows the prevalence of metabolic syndrome within individual components (Figure S1-1 to Figure S1-7). Abbreviations: DBP, diastolic blood pressure; HDL-C, high-density lipoprotein cholesterol; SBP, systolic blood pressure; TG, triglycerides; WC, waist circumference

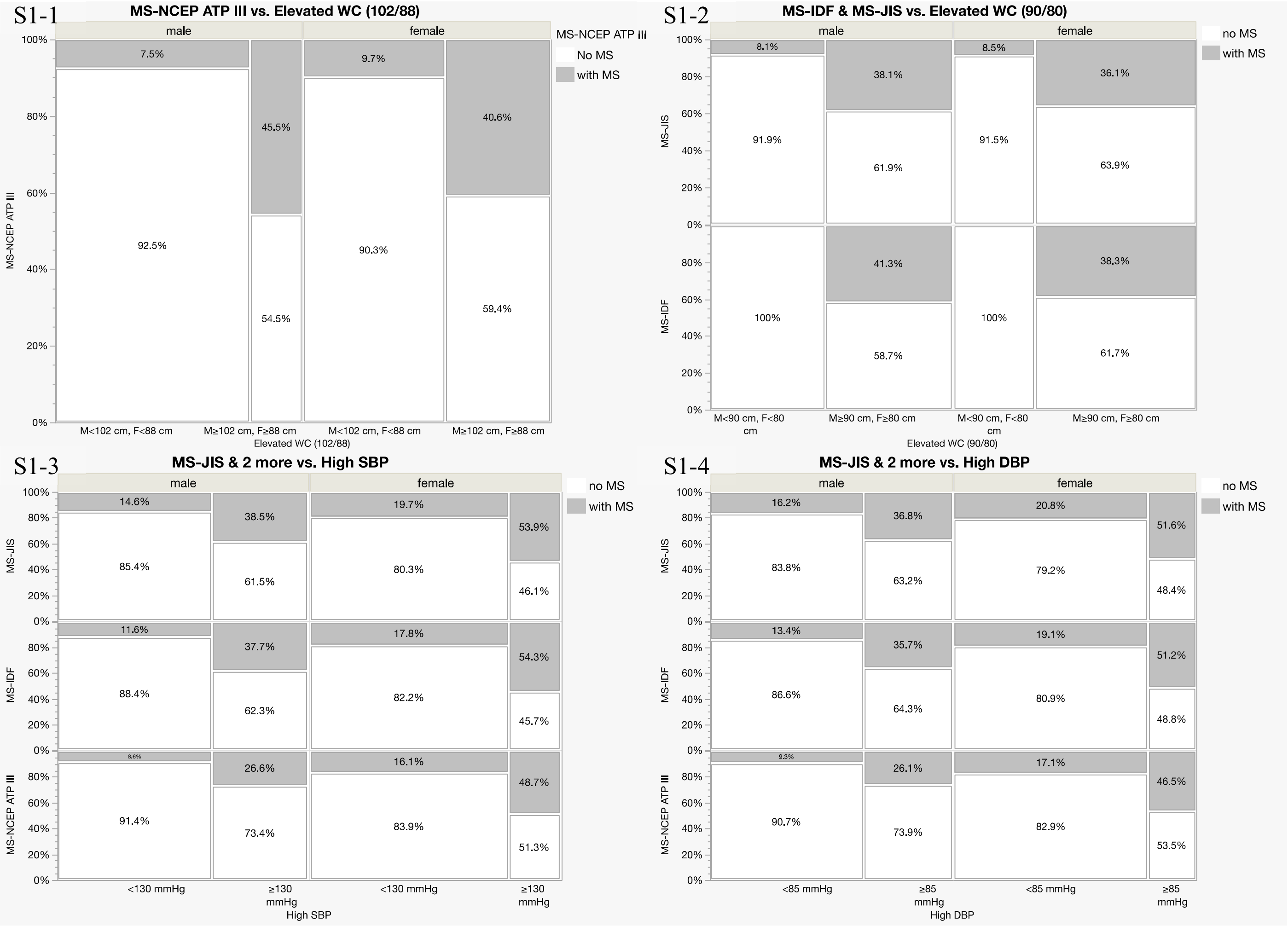

S1-5

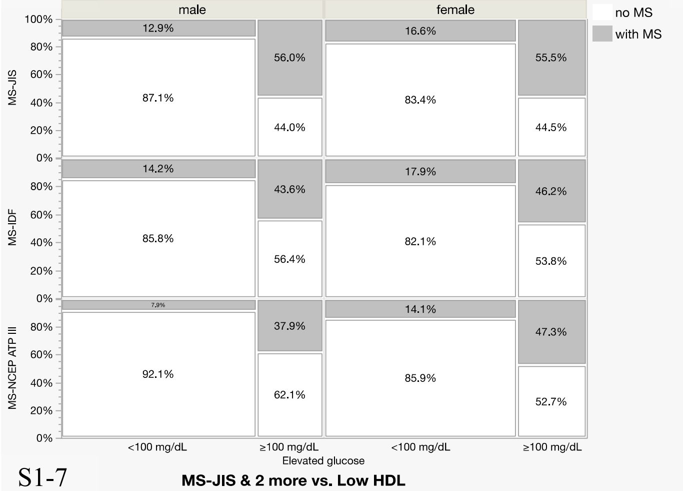

MS-JIS & 2 more vs. Elevated glucose
male
female
no MS
with MS
100%
80%
60%
40%
20%
0%
MS-JIS
MS-IDF
MS-NCEP ATP III
12.9%
87.1%
56.0%
44.0%
16.6%
83.4%
55.5%
44.5%
14.2%
85.8%
43.6%
56.4%
17.9%
82.1%
46.2%
53.8%
7.9%
92.1%
37.9%
62.1%
14.1%
85.9%
47.3%
52.7%
<100 mg/dL
≥100 mg/dL
<100 mg/dL
≥100 mg/dL
Elevated glucose

S1-6

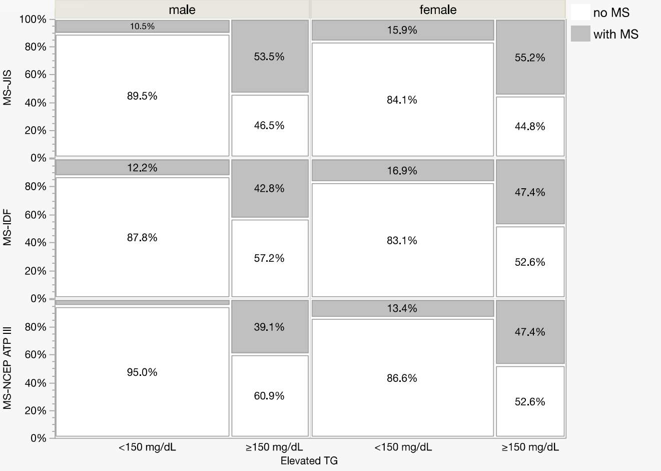

MS-JIS & 2 more vs. Elevated TG
male
female
no MS
with MS
100%
80%
60%
40%
20%
0%
MS-JIS
MS-IDF
MS-NCEP ATP III
10.5%
89.5%
53.5%
46.5%
15.9%
84.1%
55.2%
44.8%
12.2%
87.8%
42.8%
57.2%
16.9%
83.1%
47.4%
52.6%
95.0%
39.1%
60.9%
13.4%
86.6%
47.4%
52.6%
<150 mg/dL
≥150 mg/dL
<150 mg/dL
≥150 mg/dL
Elevated TG

S1-7

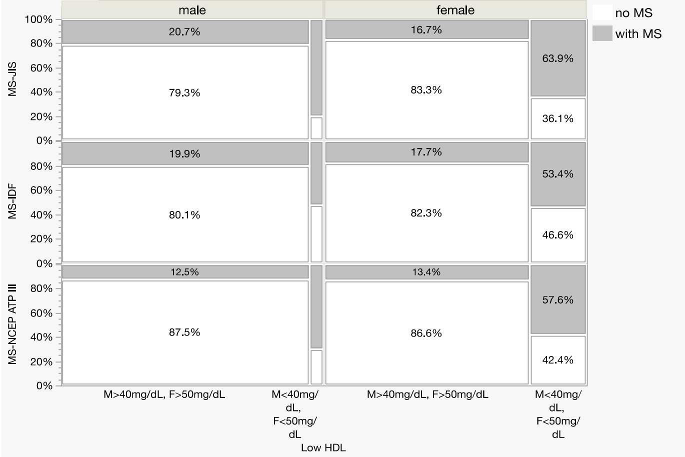

MS-JIS & 2 more vs. Low HDL
male
female
no MS
with MS
100%
80%
60%
40%
20%
0%
MS-JIS
MS-IDF
MS-NCEP ATP III
20.7%
79.3%
16.7%
83.3%
63.9%
36.1%
19.9%
80.1%
17.7%
82.3%
53.4%
46.6%
12.5%
87.5%
13.4%
86.6%
57.6%
42.4%
M>40mg/dL, F>50mg/dL
M<40mg/dL, F<50mg/dL
M>40mg/dL, F>50mg/dL
M<40mg/dL, F<50mg/dL
Low HDL
